# Unveiling the Cardioprotective Power: Liquid Chromatography–Mass Spectrometry (LC–MS)-Analyzed *Neolamarckia cadamba* (Roxb.) Bosser Leaf Ethanolic Extract against Myocardial Infarction in Rats and In Silico Support Analysis

**DOI:** 10.3390/plants12213722

**Published:** 2023-10-30

**Authors:** Raghupathi Niranjan Kumar, Dsnbk Prasanth, Praisy Gladys Midthuri, Sheikh F. Ahmad, Attuluri Venkata Badarinath, Srikanth Kumar Karumanchi, Ramanjaneyulu Seemaladinne, Rahul Nalluri, Praveen Kumar Pasala

**Affiliations:** 1 Department of Pharmacology, Santhiram College of Pharmacy, JNTUA, Nandyal 518112, Andhra Pradesh, India; niranjan3131@gmail.com (R.N.K.); praisygladys745@gmail.com (P.G.M.); 2 Department of Pharmacognosy, KVSR Siddhartha College of Pharmaceutical Sciences, Vijayawada 520010, Andhra Pradesh, India; dsnbkprasanth@gmail.com; 3 Department of Pharmacology and Toxicology, College of Pharmacy, King Saud University, Riyadh 11451, Saudi Arabia; 4 Department of Pharmaceutics, Santhiram College of Pharmacy, Nandyala 518112, Andhra Pradesh, India; avbadrinatha@gmail.com; 5 Department of Pharmaceutical Chemistry, DKSS Institute of Pharmaceutical Science & Research (for Girls), Swami-Chincholi, Bhigwan 413130, Maharashtra, India; karumanchi002@gmail.com; 6 Department of Chemistry and Biochemistry, Lamar University, Beaumont, TX 77705, USA; rseemaladinne@gmail.com; 7 Department of Pharmacy, Texas A&M University, Kingsville, TX 78363, USA; rnalluri207@gmail.com; 8 Department of Pharmacology, Raghavendra Institute of Pharmaceutical Education and Research, JNTUA, Anantapuramu 515721, Andhra Pradesh, India

**Keywords:** *Neolamarckia cadamba*, HR-LC–MS, molecular docking, myocardial infarction, oxidative stress, HMG-CoA reductase

## Abstract

*Neolamarckia cadamba* (Roxb.) Bosser, a member of the Rubiaceae family, is a botanical species with recognized therapeutic properties. It is commonly used in traditional medicine to treat cardiac ailments and other disorders. However, the precise active constituents and the potential mechanisms by which they manage cardiovascular disorders remain unclear. Therefore, this study aimed to ascertain the bioactive components and investigate their underlying mechanisms of action. *N*. *cadamba* is used to treat cardiovascular disorders using the integrated metabolomic methodology. An HPLC-QTOF-MS/MS analysis determined the potential chemicals in the *N. cadamba* leaf ethanol extract (NCEE). A thorough investigation of the NCEE samples used in this study led to the identification of 32 phytoconstituents. Of the 32 compounds, 19 obeyed Lipinski’s rule of five (RO5). A molecular docking study directed towards HMG-CoA reductase used 19 molecules. The reference drug atorvastatin indicated a binding energy of −3.9 kcal/mol, while the other substances, Cinchonain Ib and Dukunolide B, revealed binding energies of −5.7 and −5.3 kcal/mol, respectively. Both phytocompounds showed no toxicity and exhibited favorable pharmacokinetic properties. In vivo study results concluded that treatment with NCEE significantly reduced the cardiac myocardial infarction (MI) marker CK-MB and atherogenic risk indices, such as the atherogenic index plasma (AIP), cardiac risk ratio (CRR), and atherogenic coefficient (AC) in isoproterenol-induced MI rats. In MI rats, NCEE therapy significantly improved the antioxidant system of the heart tissue, as evidenced by the increased levels of GSH and SOD, lower levels of the oxidative stress marker MDA, and significantly decreased HMG-CoA activity. Additionally, electrocardiogram (ECG) signals from rats treated with NCEE resembled those treated with traditional atorvastatin to treat myocardial infarction. This study used H&E staining to show that administering NCEE before treatment reduced cardiac myocyte degeneration in rats with myocardial infarction, increased the presence of intact nuclei, and increased myocardial fiber strength. The potential cardioprotective effect observed in myocardial infarction (MI) rats treated with NCEE can be extrapolated from computational data to be caused by Cinchonain Ib.

## 1. Introduction

Myocardial infarction (MI) is a prominent global health concern, with increased morbidity and mortality rates. Myocardial damage is influenced by increased levels of inflammation, cellular proliferation, and the build-up of fibrous and adipose tissues within arterial walls [1]. Moreover, the heightened occurrence of myocardial infarction might be attributed to augmented levels of oxidative and nitrosative stress, aided by multiple well-established mechanisms [2]. Furthermore, it is essential to acknowledge that hypercholesterolemia and hypertriglyceridemia, whether occurring separately or together, substantially affect the initiation and progression of atherosclerotic plaques [3]. Previous studies have shown that lipid accumulation in arterial blood vessels results in inflammation and establishes a distinct type of atherosclerosis [4]. The primary event in atherosclerosis development is the deposition of lipid-containing cells within the arterial wall. Recent investigations have indicated that the progression of atherosclerosis is notably affected by the activity of these three pivotal enzymes. The inhibition of HMG-CoA reductase, an enzyme that regulates cholesterol biosynthesis, is crucial for cardioprotection [5]. Statins, the HMG-CoA reductase inhibitors, lower the risk of atherosclerosis and related cardiovascular events by reducing LDL cholesterol levels. In addition to cholesterol control, statins exhibit anti-inflammatory effects, improve endothelial function, stabilize atherosclerotic plaques, and possess antioxidant properties, making them a comprehensive strategy for protecting the heart [6,7].

Isoproterenol (ISO) is a potent synthetic sympathomimetic catecholamine that exerts its effects by binding β-adrenoceptors. The injection of ISO into animals results in the rapid development of infarction-like lesions. These lesions bear a striking resemblance to the morphological characteristics of coagulative myocytolysis, which is an indicator of acute myocardial infarction in humans [1,2]. Hence, scholars have employed ISO-induced myocardial damage as a paradigm to investigate the beneficial effects of various phytochemicals and synthetic drugs [3]. In recent years, the primary focus of research on cardiovascular disease therapeutics has been developing and evaluating anti-atherogenic drugs, particularly those derived from herbal sources. Owing to the minimal or negligible occurrence of adverse effects associated with herbal drugs, individuals have increasingly directed their attention toward this form of treatment [4].

*Neolamarckia cadamba*, is a medicinal plant that belongs to the Rubiaceae family. The medicinal properties and therapeutic benefits of *N*. *cadamba* have been extensively documented in ancient medicine. Charka Samhita is a classical Ayurvedic text widely regarded as one of the most authoritative and comprehensive [8]. The phytochemical analysis of *N. cadamba* has revealed the presence of several bioactive compounds. Some of the reported phytoconstituents include cadambagenic acid, cadamine, quinovic acid, β-sitosterol, cadambine, ursolic acid, prunetinoside, glucogenkwanin, prunasetin, sakuranetin, and puddumetin [9]. Reports have revealed that *N*. *cadamba* has diuretic [10], larvicidal [11,12], antidiabetic [13], antioxidant [14], anticancer, immunomodulatory [15], antiarthritic [16], anti-inflammatory, and analgesic effects [17]. Although *N*. *cadamba* is frequently used medically, insufficient data support its use in treating myocardial infarction (MI). Additionally, In silico molecular docking techniques have provided important insights into how different phytocompounds and enzymes interact. This will facilitate the development of new therapeutic strategies for treating cardiovascular diseases. The present study used In silico molecular docking analyses to investigate the potential interactions between the phytocompounds discovered in NCEE and HMG-CoA reductase, as well as to evaluate the cardioprotective effects of NCEE against ISO-induced myocardial infarction in rats, compared with those of atorvastatin, a typical inhibitor of HMG-CoA reductase in clinical use.

## 2. Results and Discussion

### 2.1. HR-LC–MS Analysis of Neolamarckia cadamba Ethanol Extract (NCEE)

**Figure 1:**
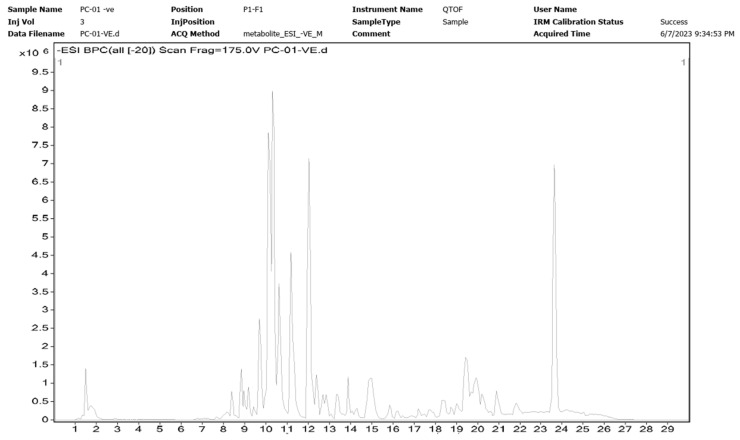
Chromatogram of the identified phytochemical constituent profiles in NCEE using HR-LC–MS.

**Figure 2:**
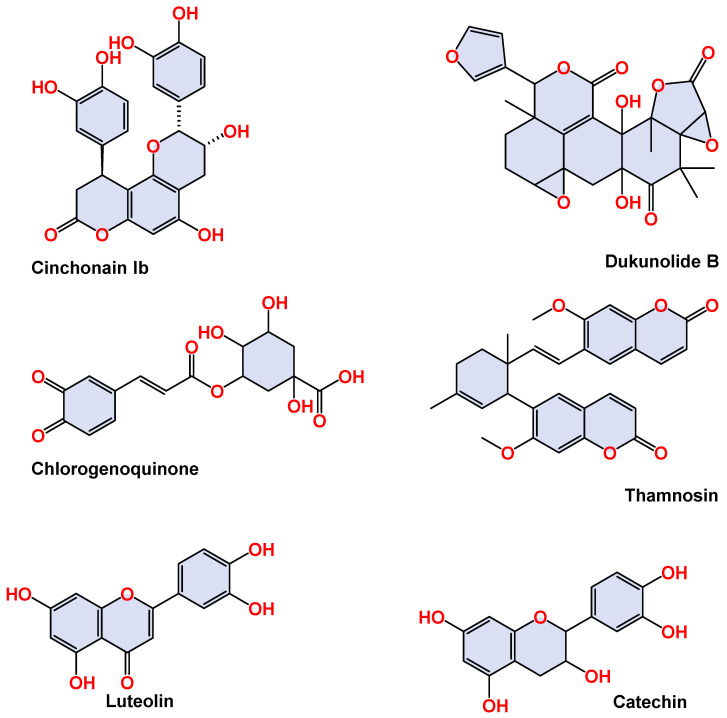
Two-dimensional representation of the top-ranked docked phytoconstituents against HMG-CoA reductase eluted from the HR-LC–MS analysis of NCEE.

**Figure 3:**
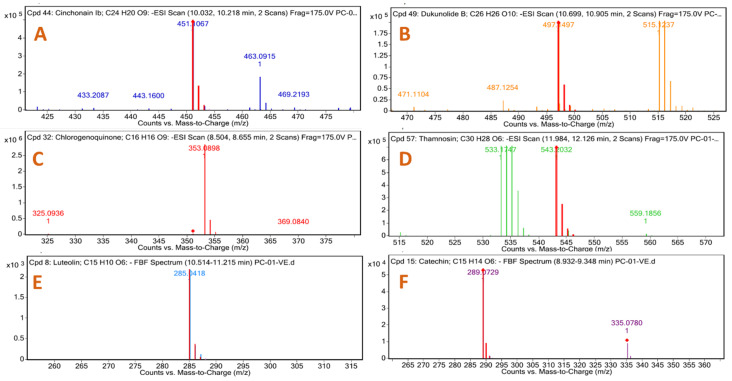
Mass spectral analysis of top-ranked docked compounds against HMG-CoA reductase eluted from NCEE: (**A**) Cinchonain Ib, (**B**) Dukunolide B, (**C**) Chlorogenoquinone, (**D**) Thamnosin, (**E**) Luteolin, and (**F**) Catechin.

The yield of NCEE was 8.53% *w*/*w*. The NCEE samples were examined using HR-LC/MS analysis to identify the phytoconstituents. The retention period, experimental *m*/*z* values, MS/MS fragments, discrepancies in the database (library), metabolite class, and suggested chemicals were used as bases for identification. Positive ionization was used to obtain mass spectrometry results. The majority of mass-to-charge (*m*/*z*) values in NCEE fall within the range of 109 to 995. The chromatogram of NCEE is shown in Figure 1. Table 1 illustrates the 32 possible phytochemical compounds identified using high-resolution liquid chromatography–mass spectrometry (HR-LC–MS) of NCEE (Figure 2 and Figure 3). The contents of Cinchonain Ib, Dukunolide B, Chlorogenoquinone, Thamnosin, Luteolin, and Catechin in NCEE were determined to be 10.08%, 1.105%, 4.222%, 4.84%, 2.02%, and 1.544%, respectively.

### 2.2. Drug Likeliness

The primary aim of this study is to identify potential therapeutic molecules with desirable drug-like properties. The compounds retrieved from NCEE were analyzed using DruLito, specifically emphasizing their drug-like characteristics. According to the data obtained from the DruLito server, of a total of 32 compounds, 19 compounds adhered to Lipinski’s rule, whereas the remaining 13 did not conform to this rule. Information on drug likeliness is presented in Table 2. The phytochemicals were subsequently subjected to molecular docking analysis to compare their outcomes with the standard drug atorvastatin (Table 2).

### 2.3. Molecular Docking Studies

**Figure 4:**
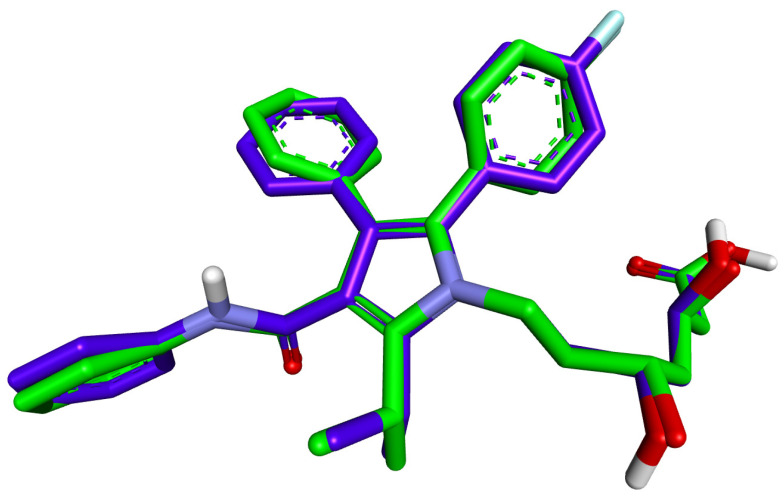
Validation of the docking algorithm by redocking the native inhibitor atorvastatin on the target human HMG-CoA reductase (PDB ID:1HWK). Green: native crystallized pose of atorvastatin; Purple: docked pose of atorvastatin.

A re-docking study validated the docking procedure before docking the ligands to the HMG-CoA reductase structure. Atorvastatin, which was present in the crystal structure at 1HWK pdb was docked to the HMGR-binding site. After validation, the RMSD value between the re-docked and co-crystallized poses was 0.9332 Å for 1HWK. These RMSD values indicated the efficiency and validity of the docking protocol (Figure 4).

**Figure 5:**
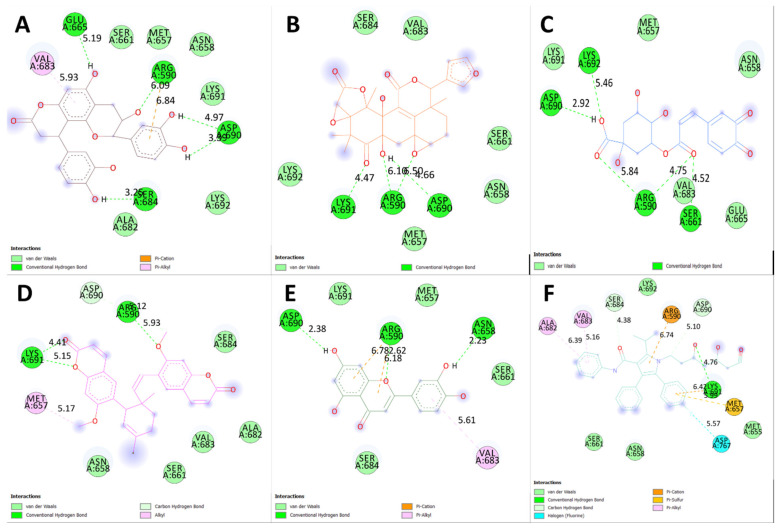
Two-dimensional interactions of phytocompounds eluted from the HR-LC–MS analysis of NCEE with HMG-CoA reductase: (**A**) Cinchonain Ib, (**B**) Dukunolide B, (**C**) Chlorogenoquinone, (**D**) Thamnosin, (**E**) Luteolin, and (**F**) Atrovastatin.

**Figure 6:**
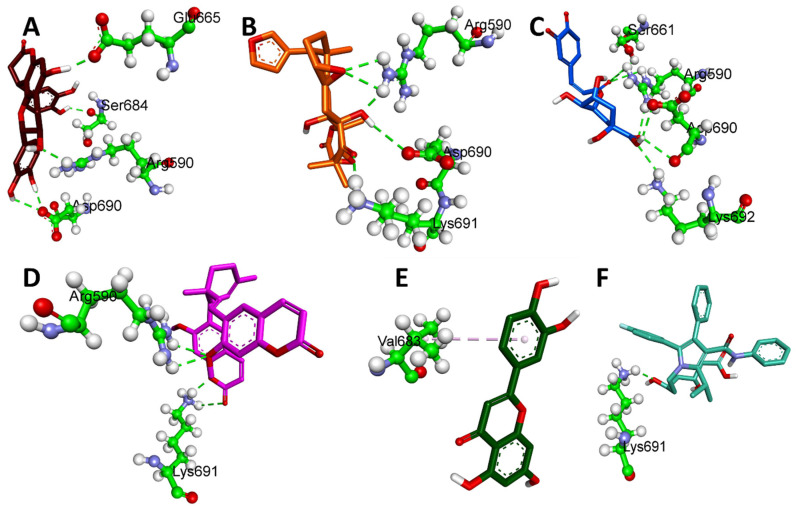
Three-dimensional H-bond interactions of phytocompounds eluted from the HR-LC–MS analysis of NCEE with HMG-CoA reductase: (**A**) Cinchonain Ib, (**B**) Dukunolide B, (**C**) Chlorogenoquinone, (**D**) Thamnosin, (**E**) Luteolin, and (**F**) Atrovastatin.

**Figure 7:**
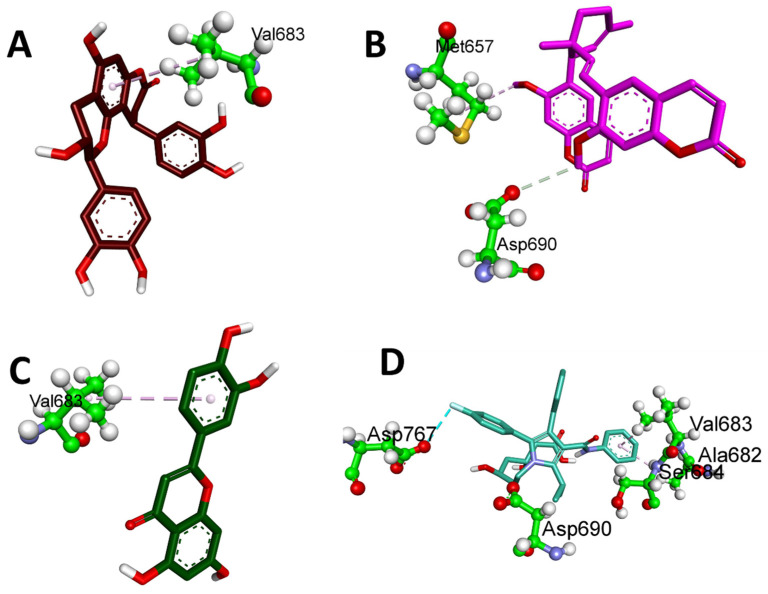
Three-dimensional hydrophobic interactions of phytocompounds eluted from the HR-LC–MS analysis of NCEE with HMG-CoA reductase: (**A**) Cinchonain Ib, (**B**) Thamnosin, (**C**) Luteolin, and (**D**) Atrovastatin.

**Figure 8:**
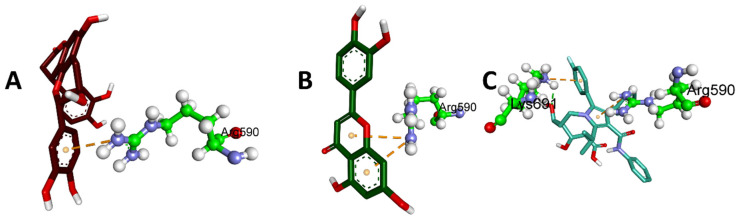
Three-dimensional electrostatic interactions of phytocompounds eluted from the HR-LC–MS analysis of NCEE with HMG-CoA reductase: (**A**) Cinchonain Ib, (**B**) Luteolin, and (**C**) Atrovastatin.

Table 3 displays the binding energies of the 19 ligands derived from NCEE, which obeyed Lipinski’s rule of five. Of the 19 compounds, 7 showed better binding affinity than the standard. This indicates that NCEE contains compounds that can combat HMG-CoA reductase activity. The 2D interactions of the ligands with their respective amino acids are presented in Figure 5, and their 3D interactions are depicted in Figure 6, Figure 7 and Figure 8.

Table 4 shows the binding affinity, ΔG (kcal/mol), of the six ligands to the target protein, the amino acids involved in binding, and the distances between them. The binding affinity measures the degree to which a ligand binds to a protein. The closer the distance between the amino acids involved in binding, the stronger the interaction.

The results showed that the binding affinities of the ligands decreased in the following order: Cinchonain Ib > Dukunolide B > Chlorogenoquinone > Thamnosin > Luteolin > Catechin > Atorvastatin. This suggests that the binding affinity of the ligands is correlated with the number and strength of the interactions between the ligand and protein (Table 4).

According to a study by Ercan et al. [18], phytocompounds from NCEE interact well with the active sites of amino acids, including Arg590, Ser661, Val683, Ser684, Asp690, and Lys691. This suggested that these phytocompounds have the potential to inhibit HMG-CoA reductase.

### 2.4. ADMET Analysis

The ADMET properties of the selected phytocompounds were evaluated using the Swiss ADME (http://www.swissadme.ch/), accessed on 22 August 2023, admetSAR (http://lmmd.ecust.edu.cn/admetsar2/), accessed on 22 August 2023 and Protox-II (https://tox-new.charite.de/protox_II/), accessed on 22 August 2023 web servers. The predicted ADMET attributes of the ligands are listed in Table 5.

To ensure the safety and efficacy of a drug, evaluating its absorption, distribution, metabolism, excretion, and toxicity (ADMET) profile is crucial [19]. This evaluation helps to identify potential issues, such as toxicity, that could result in drug withdrawal from the market. By analyzing these characteristics, researchers can determine whether a compound is likely to be absorbed, distributed, metabolized, and excreted, and whether it causes any harmful effects.

Computational methods for predicting ADMET properties, such as in silico methods, have become increasingly popular. These methods offer several advantages over traditional in vitro methods, including being faster, cheaper, and potentially more life-saving [20]. In silico processes use computer models and algorithms to predict a compound’s ADMET properties based on its chemical structure and other relevant factors without animal models. This reduces the time and expenses associated with traditional methods [21].

We evaluated the ADMET profiles of the shortlisted compounds, including drug-likeness, partition coefficients, solubility, HIA, BBB permeability, and cytochrome P450 inhibition (Table 2) [22]. Based on SwissADME, ProTox-ii, and admetSAR, we analyzed the acceptable ADME characteristics, paying particular attention to nontoxicity, as ideal drug candidates [22].

An essential property of ADMET is its ability to absorb drugs from the human gut [HIA]. The ability of drugs to be absorbed from the human gut, known as human intestinal absorption (HIA), is a crucial property of ADMET [23]. HIA plays a pivotal role in the transport of drugs to target cells [24]. A higher HIA level improves the intestinal absorption of a compound [20]. In addition to chlorogenoquinone, all compounds showed HIA values greater than 0.9, indicating good membrane permeation.

These compounds have been evaluated for their hepatotoxic, carcinogenic, and mutational potential [25]. The ProTox II results revealed that, except for Dukunolide B and Luteolin, the rest of the compounds were non-carcinogenic. They can also be used as drugs for the treatment of myocardial infarction. As these compounds cannot accumulate in the body, they are less likely to cause cancer if treated for a long time. No hepatotoxicity or cytotoxicity was observed for any of the tested compounds. ADMET studies often use these properties to analyze drug behavior [26].

### 2.5. Acute Oral Toxicity Test

The administration of 2000 mg/kg NCEE did not cause any observable signs of toxicity. The intervention did not elicit any significant alterations in the respiratory patterns, levels of consciousness, or physical mobility. Restlessness, diarrhea, coma, and convulsions were absent during the two-week follow-up period. The extract was devoid of mortality at 2000 mg/kg.

### 2.6. Effects of NCEE on Serum and Cardiac Biochemical Parameters in Rats

#### 2.6.1. Electrocardiogram Monitoring

**Figure 9:**
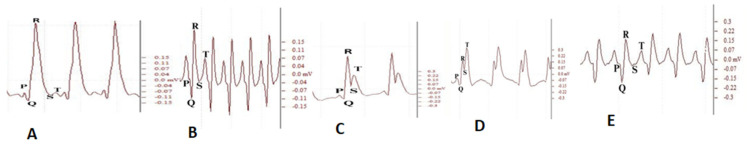
Electrocardiogram: (**A**) normal rats, (**B**) ISO, (**C**) ISO + ATV, (**D**) ISO + NCEE (200 mg/kg), and (**E**) ISO + NCEE (400 mg/kg).

**Figure 10:**
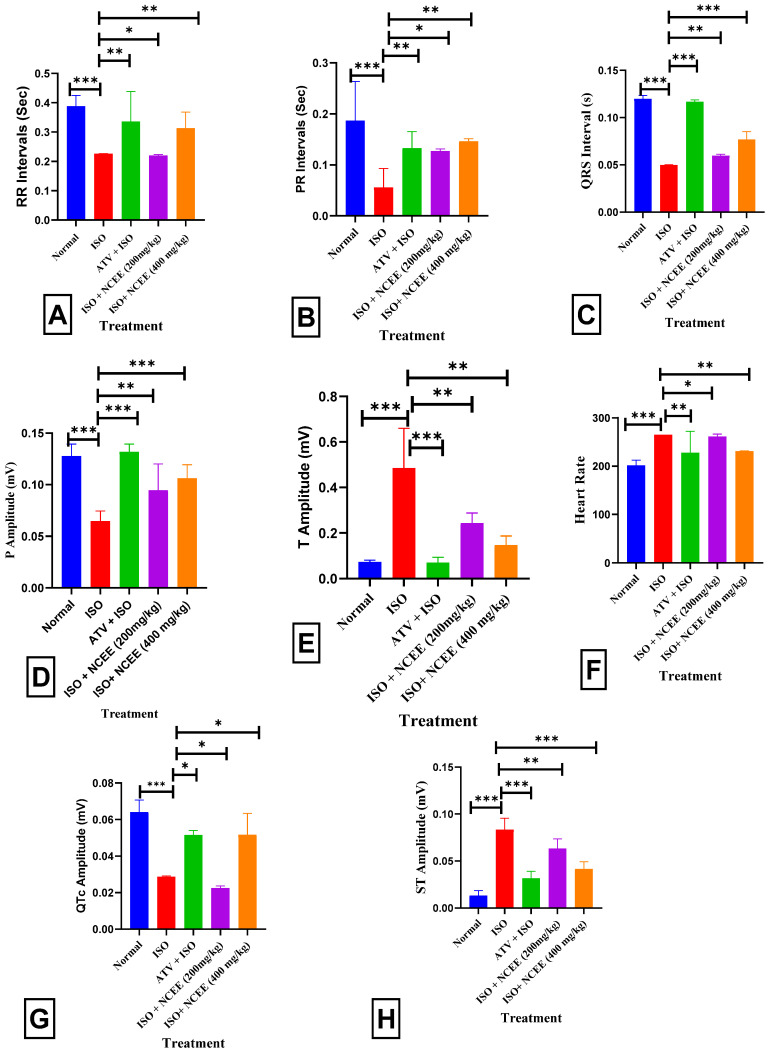
Effect of NCEE on electrocardiogram: (**A**) RR Intervals, (**B**) PR Intervals, (**C**) QRS intervals, (**D**) P Amplitude, (**E**) T Amplitude, (**F**) heart rate, (**G**) QTc amplitude, and (**H**) ST Amplitude. All results are expressed as mean ± SEM and one-way ANOVA with Dunnett’s multiple comparison tests (significance at *p* < 0.05 *, *p* < 0.01 **, *p* < 0.001 ***). The results were compared with those of the ISO group.

The ISO group (85 mg/kg) exhibited a statistically significant decrease in RR intervals (*p* < 0.001 ***), PR intervals (*p* < 0.001 ***), QRs intervals (*p* < 0.001 ***), P amplitude (*p* < 0.001 ***), and QTc amplitude (*p* < 0.001 ***), and an increase in T amplitude (*p* < 0.001 ***), as well as heart rate (*p* < 0.001 ***) and ST amplitude (*p* < 0.001 ***), compared to the normal group. In contrast, rats that received pretreatment with 400 mg/kg NCEE exhibited a statistically significant increase in the RR interval (*p* < 0.01 *), PR interval (*p* < 0.01 **), QTs interval (*p* < 0.001 ***), P amplitude (*p* < 0.001 ***), and QTc amplitude (*p* < 0.05 *), and a decrease in T amplitude (*p* < 0.01 **), as well as a decrease in heart rate (*p* < 0.01 **), when compared to the ISO group. A significant and statistically significant increase in RR intervals (*p* < 0.001 ***), PR intervals (*p* < 0.001 ***), QTs intervals (*p* < 0.001 ***), QTc amplitude (*p* < 0.05 *), and P amplitude (*p* < 0.001 ***), together with a decrease in T amplitude (*p* < 0.001 ***) and heart rate (*p* < 0.001 ***), were found in those receiving ATV treatments compared to those receiving ISO treatment. Electrocardiogram (ECG) abnormalities are frequently utilized as the principal criteria for establishing a definitive myocardial infarction (MI) diagnosis. ST-segment elevation is a significant marker of cardiomyocyte membrane injury, suggesting a potential distinction between ischemic and non-ischemic regions and the consequent impairment of cell membrane functionality [27,28]. Considering the inward sodium and calcium currents and outward potassium and chloride currents during the heart’s electrical systole, the QT interval offers insight into the heart’s functional condition [29,30]. ST elevation can determine the existence of aberrant cardiac disorders such as arrhythmias, cardiac dysfunction, and rapid cardiac collapse [28]. The electrocardiogram (ECG) of the ISO group showed significant ST-segment elevation, a significant drop in R wave amplitude, reduced RR, PR, QRs, and QTc intervals, and a substantial increase in heart rate (HR) and T amplitude. These results demonstrate the presence of isoproterenol-induced myocardial infarction (MI). The changes in electrocardiographic (ECG) patterns induced by ISO are in line with the findings of other studies [31,32]. According to Patel et al. (2010), the significant increase in heart rate (HR) in the ISO group may have been caused by ISO stimulation of the β-adrenoceptor [31]. Previous studies have provided evidence to support the notion that an elevated heart rate (HR) is causally linked to the escalation of cardiac oxygen demand, ultimately resulting in a hastened occurrence of myocardial necrosis. The administration of ISO may reduce R-wave amplitude due to the development of myocardial edema [33]. The present investigation demonstrated that groups pretreated with NCEE exhibited a significant decrease in electrocardiogram (ECG) abnormalities generated by ISO (Figure 9 and Figure 10).

#### 2.6.2. Effects of NCEE on the Serum Parameters

**Figure 11:**
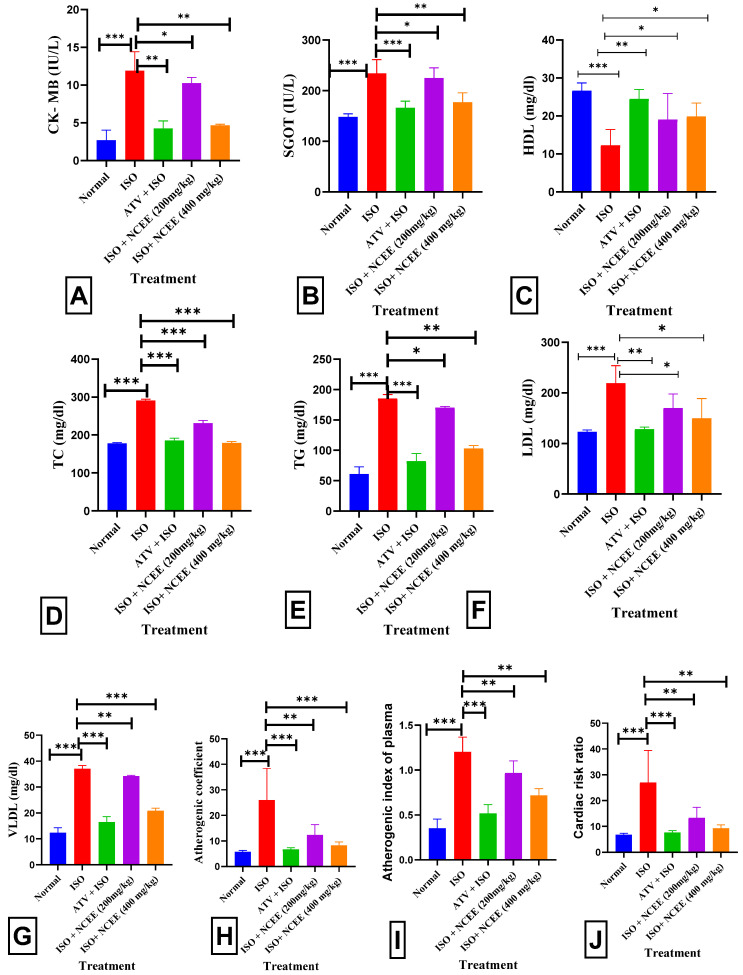
NCEE effects on serum parameters: (**A**) CK-MB levels, (**B**) SGOT, (**C**) HDL levels, (**D**) TC levels, (**E**) TG levels, (**F**) LDL levels, (**G**) VLDL levels, (**H**) AC, (**I**) AIP, and (**J**) CRR. All results are expressed as mean ± SEM and one-way ANOVA with Dunnett’s multiple comparison tests (significance at *p* < 0.05 *, *p* < 0.01 **, *p* < 0.001 ***). The results were compared with those of the ISO group.

The ISO group (85 mg/kg) exhibited a statistically significant increase in serum CK-MB (*p* < 0.001 ***), SGOT (*p* < 0.001 ***), TC (*p* < 0.001 ***), TG (*p* < 0.001 ***), LDL (*p* < 0.001 ***), VLDL (*p* < 0.001 ***), AC (*p* < 0.001 ***), AIP (*p* < 0.001 ***), and CCR (*p* < 0.001 ***), and significantly decreased HDL (*p* < 0.001 ***), when compared to the normal group. Pretreatment with 400 mg/kg NCEE resulted in a statistically significant decrease in CK-MB (*p* < 0.01 **), SGOT (*p* < 0.01 **), TC (*p* < 0.001 ***), TG (*p* < 0.01 **), LDL (*p* < 0.05 *), VLDL (*p* < 0.001 ***), AC (*p* < 0.05 *), AIP (*p* < 0.01 **), and CCR (*p* < 0.01 **), and significantly increased HDL (*p* < 0.05 **), compared to the ISO group. Creatine kinase (CK) facilitates the reversible transformation of creatine and adenosine triphosphate (ATP) into creatine phosphate and adenosine diphosphate. The myocardium comprises a dimeric enzyme consisting of two subunits, namely, M and B. Approximately 20% of the overall cardiac creatine kinase (CK) exists in the MB isoform, hence offering diagnostic specificity and sensitivity for detecting MI [34]. The findings of this study align with those of previous studies and demonstrate that ISO administration leads to necrotic myocardial injury [35,36]. The enzyme SGOT is predominantly located in the liver, heart, and muscles, and is secreted into the bloodstream in response to organ damage. According to Lofthus et al., the serum levels of SGOT activity increase within 3–8 h following the initiation of myocardial injury [37]. These levels peaked at 24 h and gradually reverted to normal throughout 3–6 days. By observing a significant increase in serum CK-MB levels in the ISO group compared with the normal group, the current investigation confirmed the incidence of MI caused by ISO. The NCEE-treated groups showed a marked reduction in the cardiac blood marker levels. These results imply that NCEE protects the heart by maintaining the structural and functional integrity of the myocyte plasma membrane and the contractile mechanism, thereby reducing the release of cardiac enzymes into the bloodstream. Lipids are crucial in atherosclerosis and hyperlipidemia and directly impact cardiovascular disease. Lipids can also alter the biological membranes’ stability, consistency, and composition. Increased blood lipid levels contribute to the development of arteriosclerosis and are significant risk factors for myocardial infarction. Myocardial infarction patients had higher levels of total cholesterol, triglycerides, LDL cholesterol, VLDL cholesterol, and lower HDL cholesterol concentrations, according to Kumar and Al-Yahya et al. [38] and Meeran, M. N. et al. [39]. In the current study, rats treated with ISO exhibited significant increases in TC, LDL, VLDL, TG, and AI levels. In addition, HDL levels were lower in these rats. However, 400 mg/kg treatment reversed these adverse effects on the lipid profile. These data demonstrated that the modulation of the lipid profile by NCEE is an essential component of its cardioprotective action (Figure 11).

**Figure 12:**
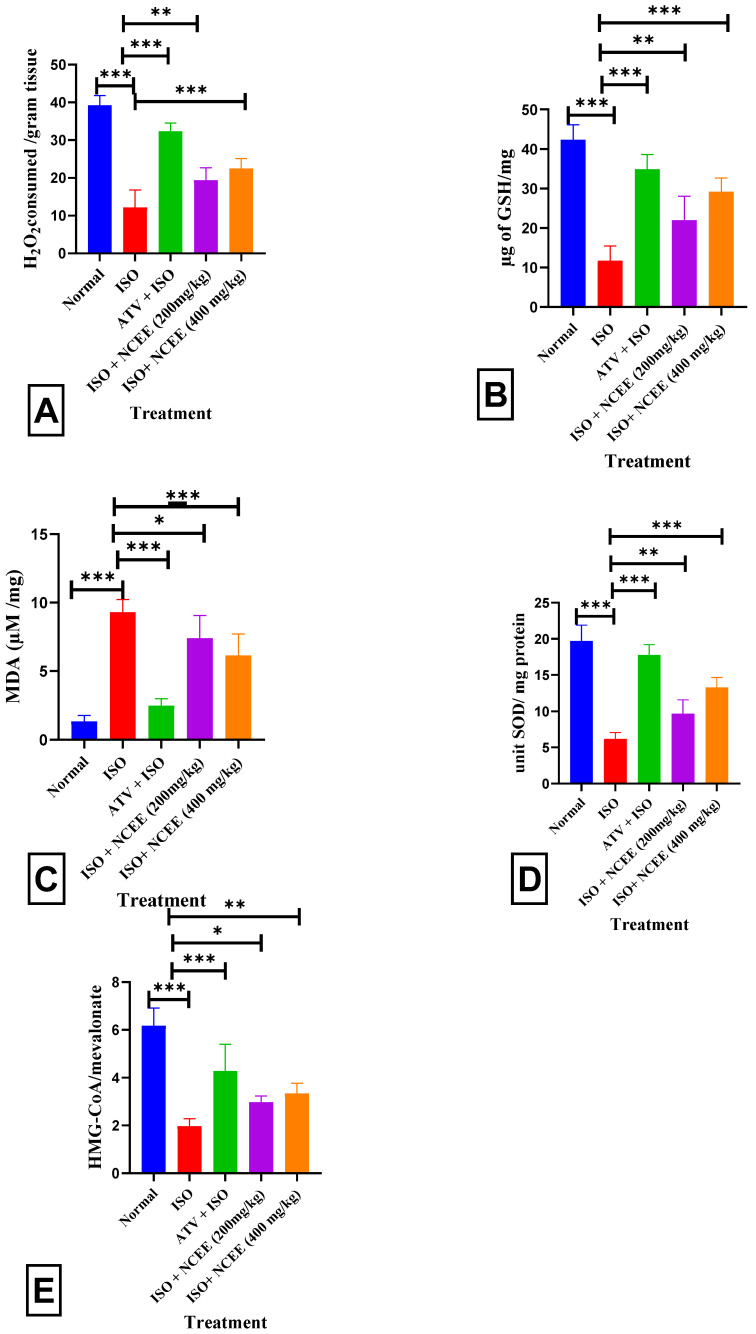
Effects of NCEE on tissue parameters. Heart tissue: (**A**) CAT activity, (**B**) GSH level, (**C**) MDA level, and (**D**) SOD activity; liver tissue: (**E**) HMG-CoA activity. All results are expressed as mean ± SEM and one-way ANOVA with Dunnett’s multiple comparison tests (significance at *p* < 0.05 *, *p* < 0.01 **, *p* < 0.001 ***). The results were compared with those of the ISO group.

TIe ISO group exhibited a notable decrease in cardiac antioxidant levels, specifically CAT (*p* < 0.001 ***), GSH (*p* < 0.001 ***), and SOD (*p* < 0.001 ***). Additionally, an elevation in oxidative stress was observed, as indicated by an increase in MDA levels (*p* < 0.001 ***). Pretreatment with NCEE (400 mg/kg) resulted in a significant increase in CAT (*p* < 0.001 ***), GSH (*p* < 0.001 ***), and SOD (*p* < 0.001 ***), as well as a decrease in MDA (*p* < 0.001 ***), compared to the ISO group. Liver HMG-CoA reductase activity was increased as reflected by a decrease in the HMG-CoA/mevalonate ratio (*p* < 0.001 ***) in ISO-treated rats compared to normal rats (Figure 12). The formation of the reactive hydroxyl radical (OH•), which acts as a catalyst for lipid peroxidation and damages and disrupts cellular membranes, is frequently blamed for the incidence of ISO-induced myocardial infarction (MI) [40]. Antioxidant enzymes, such as superoxide dismutase (SOD) and catalase (CAT), are critical players in the initial defense mechanisms within cells to combat oxidative stress caused by diverse stressors. Our study showed that the myocardial SOD and CAT activities were significantly lower in the model group than in the control group. SOD and CAT enzyme activities were lower in ISO-induced MI, which may be related to an increase in the generation of reactive oxygen species, specifically superoxide and hydrogen peroxide. Therefore, oxidative stress may suppress SOD and CAT activity [41]. Reactive oxygen species (ROS) accumulate due to GSH inhibition, making cardiomyocytes more susceptible to oxidative injury [42,43]. In contrast, 400 mg/kg NCEE administration resulted in a notable increase in enzymatic activity and GSH levels. These findings confirmed that the antioxidative properties of NCEE play a crucial role in its cardioprotective effects.

#### 2.6.3. Liver HMG-CoA Analysis

The assessment of HMG-CoA reductase activity using an indirect approach demonstrated that the ISO-induced disease group exhibited a significant (*p* < 0.001 ***) reduction in the HMG-CoA/mevalonate ratio (Figure 12E). This reduction suggests an elevation in cholesterol synthesis compared to the normal group. In the groups pretreated with 400 mg/kg NCEE, a statistically significant increase (*p* < 0.01 **) in the HMG-CoA/mevalonate ratio was observed. This finding suggests that NCEE can potentially decrease the overall activity of HMG-CoA reductase compared to the ISO group. Following the administration of ISO, the evaluation of the blood lipid profile revealed substantial dysregulation characterized by a marked elevation in triglyceride, cholesterol, LDL-C, and VLDL-C levels, accompanied by a considerable decrease in HDL-C values. These findings suggest a hyperlipidemic effect possibly resulting from perturbations in lipid metabolism in the treatment of ISO. This finding suggests a potential direct association with myocardial infarction [44]. The higher the ratio, the lesser the mevalonate formed, indicating a reduction in HMG-CoA reductase activity and reduced cholesterol synthesis [6,45]. The hypolipidemic activity of NCEE may result from its HMG-CoA reductase activity, which controls cholesterol synthesis. This result is similar to the hypolipidemic and HMG-CoA reductase activity-reducing capability of sulfated polysaccharides from *Padina tetrastromatica* [6].

### 2.7. Histopathological Studies

**Figure 13:**
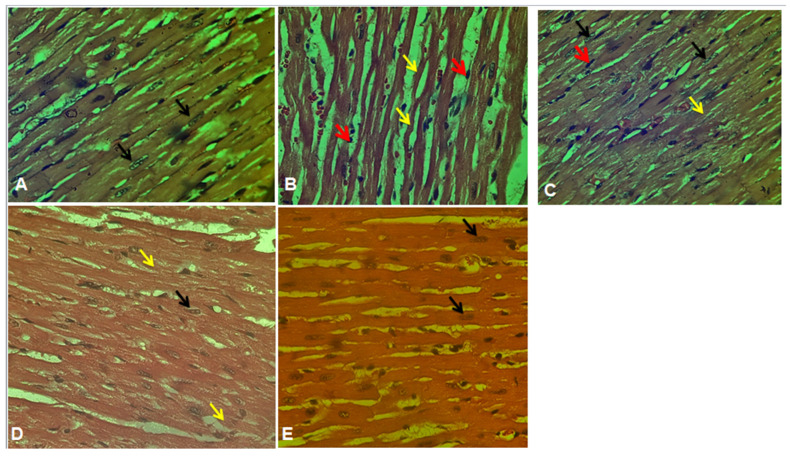
Histopathological examination of the heart in experimental groups of rats. (**A**) Control group with the standard histological architecture of cardiac myocytes with prominent nuclei (black arrow). (**B**) The ISO group showed a marked degeneration of cardiac muscle fibers; extensive damage of cardiac myocytes (yellow arrow); loss of cytoplasmic striation of myocytes with shrinkage, elongation, and disarrangement of nuclei; and marked inflammatory cell infiltration, mainly neutrophils; myocytes showed an absence of nuclei (red arrow). (**C**) The ATV-treated group showed compact, uniformly arranged myocardial fibers, few areas of chronic inflammatory cells (red arrow), myocytes with uniform nuclei, and striations (black arrow). (**D**) Group IV NCEE (200 mg/kg, per oral) showed an appearance of damage of cardiac myocytes (yellow arrow), few cardiac myocytes with prominent nuclei (black arrow). (**E**) Group V NCEE (400 mg/kg, per oral) showed compact, uniformly arranged myocardial fibers, with uniformly and prominently placed nuclei (black arrow). A significant loss of wave-like cardiac fibers was observed, and fewer inflammatory cells appeared in some areas than in group IV NCEE (200 mg/kg)-treated rats.

The histopathological analysis of the ISO group demonstrated cardiac muscle fiber degradation and significant damage to cardiac myocytes (Figure 13), which aligns with previous findings reported by Qudus et al. (2021) [46]. The myocardial fibers in the NCEE (400 mg/kg) group exhibited a practically normal architecture, indicating a minimal degree of deterioration. These findings provide further evidence to support the cardioprotective action of NCEE.

## 3. Materials and Methods

### 3.1. Plant Material and Extraction

The *N. cadamba* specimen was collected and later identified in the Department of Botany at the PSC & KVSC Government College in Nandyal, Andhra Pradesh, India. A voucher specimen was deposited at the herbarium of the same faculty and assigned number PSCKVSC/BOT/0009. The leaves were then shade-dried, ground, powdered, and extracted using ethanol. The extract was subsequently dried under a vacuum in a rotary evaporator.

### 3.2. Identification of Bioactive Compounds by High-Resolution Liquid Chromatography–Mass Spectrometry (HR-LC–MS)

High-resolution liquid chromatography–mass spectrometry (HR-LC–MS) analysis was performed on the NCEE sample using a ChipCube G6550A iFunnel Q-TOF mass spectrometer equipped with an electrospray ionization source. Phytochemicals were separated using a Hypersil GOLD C-18 column (2.1 × 100 mm, three μm particle size) as the stationary phase. The gradient mobile phases of “solvent A” (100% water) and “solvent B” (100% methanol) were used at a flow rate of 300 μL/min. The injection volume of NCEE was three μL at an injection speed of 100 μL/min with a 5.0 sample flush-out factor. The gradient was started with 95:5 (H_2_O/CH_3_OH) for 1 min, changed to 0:100 (H_2_O/CH_3_OH) for 25 min, and returned to 95:5 (H_2_O/CH_3_CN) for 31 min. The iFunnel MS Q-TOF instrument segment was maintained at a gas flow rate of 13 L/min, temperature of 250 °C, gas flow rate of 11 L/min, sheath gas flow rate of 300 °C, and a nebulizer gas flow pressure of 35 psi. The acquisition method was set to the MS mode with a minimum range of 120 (*m*/*z*) and a maximum of 1200 (*m*/*z*), scanning at a rate of 1 spectrum/s. The analysis was conducted at the Sophisticated Analytical Instrument Facility (SAIF) of the Indian Institute of Technology, Bombay (IIT Bombay), India [47,48].

### 3.3. In-Silico Studies

#### 3.3.1. Drug-Likeliness

To assess the drug-like properties of the phytoconstituents identified in NCEE, a drug-likeness tool (DruLito) was employed. DruLito evaluated the chemical compounds based on Lipinski’s rule of five, which examines parameters such as molecular weight, log P, and the number of hydrogen bond donors and acceptors. This analysis provides insights into the likelihood that the identified phytoconstituents possess drug-like properties [49].

#### 3.3.2. Molecular Docking

**Figure 14:**
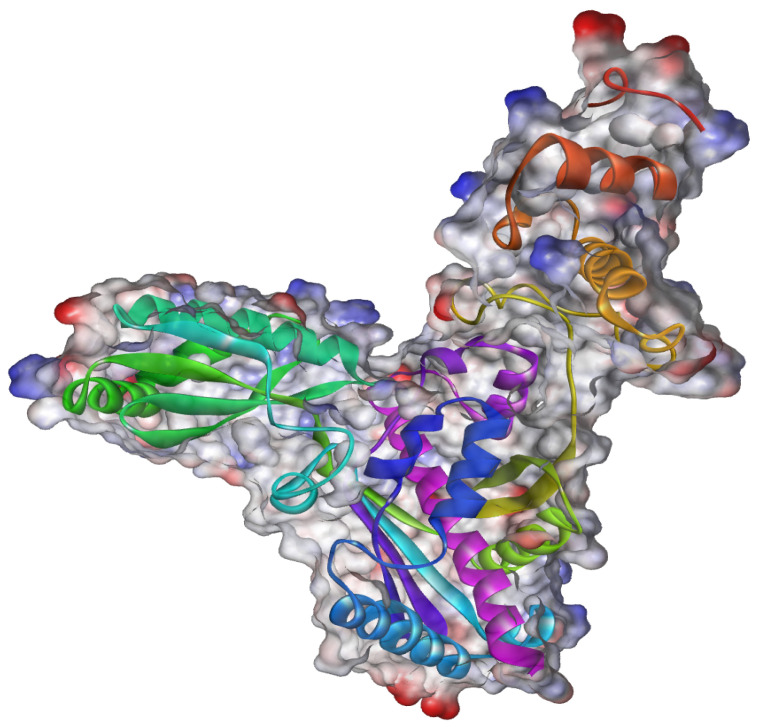
Three-dimensional ribbon-type representation of the complex of the catalytic portion of human HMG-CoA reductase with atorvastatin (PDB: 1HWK).

The docking study was implemented using Auto Dock Vina, and relevant input files for Auto Dock Vina were generated using the Auto Dock program. Human HMG-CoA reductase (PDB ID:1HWK) (Figure 14) [50] was used as a target for this study. The RSCB protein data bank provided crystallized structures, and PubChem provided 3D structural information on the ligands. Incorporating polar hydrogen atoms and gesture charges is necessary to prepare files through Auto Dock. Table 6 presents the coordinates of the grid box and grid box size. Vina was implemented using a shell script supplied by Auto Dock Vina developers. The strength of the bond between the ligand and receptor is represented as a negative score (kcal/mol). The Autodock Vina script produced nine distinct ligand positions with different binding energies for each ligand. A Perl script obtained the ligand with the highest binding affinity for the docked complexes [51].

#### 3.3.3. ADMET Analysis

Furthermore, the absorption, distribution, metabolism, excretion, and toxicity (ADMET) prediction tool ADMETSAR was used to evaluate the potential pharmacokinetic properties and toxicity risks associated with the identified phytoconstituents. ADMETSAR utilizes computational models to predict aqueous solubility, blood–brain barrier permeability, cytochrome P_450_ inhibition, hepatotoxicity, and mutagenicity. This analysis aids in assessing the overall drug-like characteristics and potential safety concerns of the phytoconstituents. The ADMETSAR results provide valuable insights into the ADME properties and potential risks associated with the identified compounds, guiding further investigations for developing safe and effective cerebroprotective drugs [52].

### 3.4. Acute Toxicity

Based on earlier works on acute toxicity studies of NCEE on Wistar rats, according to OECD—423 guidelines, the different doses of NCEE were administered up to 4000 mg/kg b.w. (p.o.) and there is no evidence of toxicity reported. According to the OECD—423 guidelines, in the present study, 1/10th and 1/20th doses are preferred as high and low doses [53].

### 3.5. Induction of MI

Wistar rats (8 weeks old, both sexes, weight average 220–225 gm) were subjected to subcutaneous injection of isoproterenol at a dose of 85 mg/kg, administered at 24 h intervals over two consecutive days, inducing specific alterations in biochemical markers, histological observations, and electrocardiogram (ECG) readings that are indicative of myocardial infarction (MI) [54,55,56].

### 3.6. Experimental Procedure

Thirty rats were randomly assigned to five groups of six rats each. The treatment regimen was as follows. Experimental protocols were carried out with the approval of Institutional Animal Ethics Committee protocol number (1519/po/Re/S/11/CPCSEA/2022/004)

1. Normal group: rats received two subcutaneous injections of normal saline (vehicle of ISO) in a volume of 1.5 mL/kg, with one injection per day on days 8 and 9.
2. In the ISO group, rats received two injections of ISO (85 mg/kg/day, s.c.) at a volume of 1.5 mL/kg, with one injection per day on days 8 and 9, to induce acute MI.
3. ATV + ISO group: rats received atorvastatin (10 mg/kg, per oral) for nine days, followed by two injections of ISO (85 mg/kg/day, s.c.), with one injection per day on days 8 and 9.
4. NCEE (200 mg/kg) + ISO group: rats were treated with NCEE at a dose of 200 mg/kg/day (p.o.) for nine days, followed by two injections of ISO (85 mg/kg/day, s.c.), with one injection per day on days 8 and 9.
5. NCEE (400 mg/kg) + ISO group: rats were treated with NCEE at a dose of 400 mg/kg/day (p.o.) for nine days, followed by two injections of ISO (85 mg/kg/day, s.c.), with one injection per day on days 8 and 9.

### 3.7. Electrocardiogram Monitoring

After administering the second dose of ISO, electrocardiograms (ECGs) were measured for all experimental groups after 24 h. The rats were administered anesthesia with Ketamine (60 mg/kg) and Xylazine (9 mg/kg) until complete anesthesia was achieved. Subcutaneous attachment of ECG electrodes was performed on the paws and chest of the rats while they were in a supine position, followed by connection to the Polyrite D (Polygraph Digital, RMS, India). Subsequent electrocardiogram (ECG) parameters were analyzed. The RR, PR, and QRS intervals, as well as the P and T amplitudes, heart rate, and QTc amplitude, are important parameters used in the analysis of cardiac activity.

### 3.8. Collection of Blood and Tissue Samples

After the ECG recordings, a clean, sterile capillary tube was placed into the eye’s inner canthus to draw blood samples from the orbital sinus (retro-orbital plexus) under anesthesia. After cervical dislocation, the rats were slaughtered, and blood was clotted for 20 min. The samples were then centrifuged for 15 min at 4000 rpm. Serum samples were separated to estimate lipid profiles: total cholesterol (TC), triglycerides (TG), HDL, SGOT, and the cardiac marker CK-MB. Atherogenic indices were determined using lipid profile data: atherogenic index of plasma (AIP), cardiac risk ratio (CRR), and atherogenic coefficient (AC) [57]. After sacrifice, the hearts were removed free of connective tissue, cleaned in ice-cold phosphate-buffered saline, and homogenized, and the supernatant was used for the estimation of antioxidant parameters such as catalase (CAT), reduced glutathione (GSH) [58], superoxide dismutase (SOD), and the stress parameter malondialdehyde (MDA). HMG-CoA (β-Hydroxyl ß-methylglutaryl-CoA) reductase activity was assessed in the liver tissue homogenate by measuring the ratio of HMG-CoA to mevalonate [59].

### 3.9. Histopathological Studies

The myocardial tissue from the excised heart was immediately fixed in a 10% buffered neutral formalin solution for histopathological studies. The fixed tissues were embedded in paraffin, and serial sections were cut. Each section was stained with hematoxylin and eosin (H&E stain). The cells were examined under a light microscope, and photomicrographs were obtained [60].

### 3.10. Statistical Analysis

All data are expressed as the mean ± S.E.M. Statistical significance between more than two groups was tested using one-way ANOVA, followed by Dunnett’s multiple comparison test using a computer-based fitting program (Prism Graph Pad 8.5). *p*-values of less than 0.05 *, 0.01 **, and 0.001 *** were considered statistically significant.

## 4. Conclusions

In summary, the results of the present study have demonstrated that the analysis of NCEE using HR-LC–MS has revealed the presence of significant groups of phytochemicals that may potentially contribute to the inhibition of HMG-CoA reductase, thereby offering potential therapeutic applications for myocardial infarction. Based on the outcomes of the molecular docking, Cinchonain Ib, Dukunolide B, and Chlorogenoquinone exhibited higher binding affinities compared to atorvastatin. Further experimental investigations are mandatory to establish their effectiveness. The current study’s in vivo findings indicate that NCEE possesses potential antioxidants that could be accountable for its advantageous impact on cardiovascular illness.

## Figures and Tables

**Table 1:** Phytochemical compounds identified in NCEE using HR-LC–MS.

tR (min)	*m*/*z*	Error (ppm)	Molecular Formula	Molecular Weight	Height	Base Peak	Identification
1.407	239.0782	−3.36	C_7_H_14_O_6_	194.0797	7259	191.0567	D-Pinitol
1.482	191.0558	1.62	C_7_H_12_O_6_	192.0631	6,939,261	191.0558	Quinic acid
1.534	133.0148	−4.86	C_4_H_6_O_5_	134.0222	19,152	191.0569	L-Malic acid
5.805	169.0143	−0.66	C_7_H_6_O_5_	170.0216	3300	197.8084	Gallic acid
7.643	109.0295	0.56	C_6_H_6_O_2_	110.0367	35,876	355.104	Resorcinol
7.643	153.0196	−2.11	C_7_H_6_O_4_	154.0269	71,074	197.8085	2,6-dihydroxybenzoic acid
8.622	351.0731	−2.49	C_16_H_16_O_9_	352.0803	1,261,779	191.0571	Chlorogenoquinone
8.874	593.1546	−5.48	C_27_H_30_O_15_	594.1617	1,368,207	353.0689	Biorobin
8.975	253.0718	−0.14	C_10_H_10_O_4_	194.0579	6547	191.0571	Vanillin acetate
9.046	177.0199	−3	C_9_H_6_O_4_	178.0271	28,346	191.0571	5,7-Dihydroxychromone
9.066	399.1312	−4.71	C_17_H_22_O_8_	354.1331	468,368	191.0568	Fusarenone X
9.109	179.0357	−3.82	C_9_H_8_O_4_	180.0429	290,533	191.0571	4-Hydroxyphenylpyruvic acid (HPPA)
9.109	289.0729	−3.44	C_15_H_14_O_6_	290.08	162,667	191.0571	Catechin
9.569	121.0297	0.92	C_7_H_6_O_2_	122.0367	10,887	609.15	Benzoic acid
9.785	739.1713	1457.58	C_34_H_33_NO_15_	694.1732	845,632	177.0188	Dexylosylbenanomicin A
9.979	463.0913	−6.47	C_21_H_20_O_12_	464.0985	486,727	300.0288	Myricitrin
10.032	163.0409	−4.77	C_9_H_8_O_3_	164.0481	29,719	565.2001	4-Hydroxycinnamic acid
10.578	285.0418	−4.84	C_15_H_10_O_6_	286.0491	7696	515.1239	Luteolin
10.619	381.1218	5.42	C_15_H_28_O_7_P_2_	382.129	394,523	135.0458	2-cis,6-trans-farnesyl diphosphate
10.629	529.2234	−7.5	C_27_H_34_N_2_O_9_	530.2304	698,376	295.1093	3-α(S)-Strictosidine
10.819	497.1497	−9.02	C_26_H_26_O_10_	498.1571	669,685	109.0298	Dukunolide B
10.848	625.125	−8.1	C_30_H_26_O_15_	626.1322	564,414	300.0297	6″-Caffeoylhyperin
10.905	447.0937	−0.74	C_21_H_20_O_11_	448.1009	50,786	451.1075	Kaempferol 7-O-glucoside
11.235	451.1073	−8.49	C_24_H_20_O_9_	452.1146	4,076,514	341.0697	Cinchonain Ib
12.068	543.2032	−1.18	C_30_H_28_O_6_	484.1892	1,446,440	265.1011	Thamnosin
12.076	995.4003	−18.71	C_45_H_61_CoN_6_ O_12_	936.3855	405,084	265.1003	Cob(I)urinate a,c diamide
12.35	613.1406	−1.11	C_20_H_31_N_4_O_16_ P	614.1479	444,048	341.0697	CMP-N-acetylneuraminic acid
15.118	503.3421	−8.52	C_30_H_48_O_6_	504.3494	710,989	503.3415	Protobassic acid
15.781	501.3266	−9.08	C_30_H_46_O_6_	502.334	388,829	435.2933	Esculentic acid (Phytolacca)
19.378	617.3906	1809.59	C_33_H_53_NO_6_	558.3769	494,114	163.0418	gamma-Chaconine
19.551	615.3201	−4.64	C_33_H_46_O_8_	570.3219	1,266,231	163.0419	Avermectin B1b aglycone
20.291	647.4022	−10.26	C_40_H_56_O_7_	648.4093	945,095	193.0534	trans-3-Feruloylcorosolic acid
23.745	455.3595	−13.7	C_30_H_48_O_3_	456.3666	5,285,974	455.3583	Ursolic acid

**Table 2:** Drug-likeness properties of various compounds eluted from HR-LC–MS of NCEE.

Sr. No.	Compound	MW	logp	HBA	HBD	TPSA	AMR	Lipinski’s Rule Violated
1	Benzoic acid	115.99	0.982	2	0	17.07	36.96	No
2	5,7-Dihydroxychromone	171.98	0.554	4	0	26.3	48.72	No
3	4-Hydroxycinnamic acid	155.98	0.751	3	0	17.07	48.8	No
4	Luteolin	275.97	1.486	6	0	26.3	81.76	No
5	Gallic acid	163.97	0.964	5	0	17.07	41.77	No
6	4-Hydroxyphenylpyruvic	171.98	−0.229	4	0	34.14	48.46	No
7	Catechin	275.97	0.852	6	0	9.23	81.07	No
8	Vanillin acetate	183.98	0.78	4	0	52.6	53.72	No
9	Resorcinol	103.99	0.654	2	0	0	34.17	No
10	D-Pinitol	179.97	−1.797	6	0	9.23	40.53	No
11	2,6-dihydroxybenzoic acid	147.98	1.045	4	0	17.07	40.17	No
12	Ursolic acid	407.98	8.954	3	0	17.07	132.26	Yes
13	Thamnosin	455.97	4.775	6	0	71.06	148.54	No
14	Quinic acid	179.97	−1.979	6	0	17.07	38.88	No
15	Kaempferol 7-O-glucoside	427.94	0.18	11	0	44.76	114.53	Yes
16	Chlorogenoquinone	335.95	−2.011	9	0	77.51	82.81	No
17	Biorobin	563.92	−1.083	15	0	63.22	145.57	Yes
18	Fusarenone X	331.96	−0.967	8	0	65.13	81.98	No
19	Dexylosylbenanomicin A	661.93	−0.705	16	0	95.97	175.16	Yes
20	Myricitrin	443.94	1.15	12	0	44.76	116.37	Yes
21	2-cis-6-trans-farnesyl diphosphate	353.91	1.888	7	0	72.22	96.1	No
22	Dukunolide B	471.95	−1.089	10	0	103.96	115.45	No
23	6″-Caffeoylhyperin	599.92	1.378	15	0	71.06	163.06	Yes
24	Cinchonain Ib	431.95	1.18	9	0	35.53	125.14	No
25	Thamnosin	455.97	4.775	6	0	71.06	148.54	No
26	CMP-N-acetylneuraminic acid	582.9	−6.358	20	0	130.61	121.07	Yes
27	Protobassic acid	455.97	5.874	6	0	17.07	140.37	Yes
28	Esculentic acid (Phytolacca)	455.97	5.855	6	0	34.14	137.52	Yes
29	gamma-Chaconine	505.97	5.035	7	0	21.7	147.36	Yes
30	Avermectin B1b aglycone	523.96	1.137	8	0	53.99	152.72	Yes
31	trans-3-Feruloylcorosolic acid	591.96	9.313	7	0	52.6	187.26	Yes
32	Ursolic acid	407.98	8.954	3	0	17.07	132.26	Yes

**Table 3:** Molecular docking of eluted compounds from HR-LC–MS of NCEE with HMG-CoA reductase (PDB:1HWK).

Compound	Binding Energies (kcal/mol)
Cinchonain Ib	−5.7
Dukunolide B	−5.3
Chlorogenoquinone	−4.9
Thamnosin	−4.9
Luteolin	−4.8
Catechin	−4.7
Fusarenone X	−4.2
2-*cis*-6-*trans*-farnesyl diphosphate	−3.9
Quinic acid	−3.9
Vanillin acetate	−3.8
4-Hydroxyphenylpyruvic	−3.7
5,7-Dihydroxychromone	−3.7
D-Pinitol	−3.7
4-Hydroxycinnamic acid	−3.6
Gallic acid	−3.6
2,6-dihydroxybenzoic acid	−3.5
Benzoic acid	−3
Resorcinol	−2.8
Atorvastatin	−3.9

**Table 4:** Binding energies and interaction details of eluted compounds from HR-LC–MS of NCEE with HMG-CoA reductase (PDB:1HWK).

Ligands	Binding Affinity, ΔG (kcal/mol)	Amino Acids Involved and Distance (Å)		
		Hydrogen-Bond Interactions	Hydrophobic Interactions	Electrostatic Interactions
Cinchonain Ib	−5.7	ARG A:590 (6.09), GLU A:665 (5.19), SER A:684 (3.25), ASP A:690 (3.39, 4.47)	VAL A:683 (5.93)	ARG A:590 (6.84)
Dukunolide B	−5.3	ARG A:590 (6.10, 6.50), ASP A:690 (4.66), LYS A:691 (4.47)	-	-
Chlorogenoquinone	−4.9	ARG A:590 (2.34, 4.75), ASP A:690 (2.92), SER A:661 (2.95), LYS A:692 (2.66)	-	-
Thamnosin	−4.9	ARG A:590 (5.93), LYS A:691 (5.17)	MET A:657 (5.17), ASP A:690 (5.12)	-
Luteolin	−4.8	ARG A:590 (6.18), ASP A:690 (2.94), ASN A:658 (3.51)	VAL A:683 (5.61)	ARG A:590 (5.93, 6.78)
Catechin	−4.7	ARG A:590 (4.70), SER A:661 (4.17), ASN A:658 (4.53), ASP A:690 (3.93)	-	ARG A:590 (7.42, 7.64)
Artovastatin	−3.9	LYS A:691 (4.76)	ASP A:767 (5.57), ALA A:682 (6.39), VAL A:683 (5.16), SER A:684 (4.38), SP A:690 (5.10)	ARG A:590 (6.74), MET A:657 (5.99, 6.42)

**Table 5:** ADMET analysis of selected phytoconstituents from the LC–MS analysis of NCEE based on molecular docking studies.

Phytocompounds	Swiss ADME								ADMETSAR							PROTOX-II			
	log P o/w	Water Solubility	GI Absorption	Lipinski’s Rule	Veber’s Rule	PAINS Alert	TPSA	Lead Likeliness	HIA	CaCO2	CYP1A2	CYP2C19	CYP2C9	CYP2D6	LD50 (mg/kg)	Hepatotoxicity	Carcinogenicity	Mutagenicity	Cytotoxicity
Cinchonain Ib	1.72	Moderately soluble	Low	Yes	No	1	156.91	No	0.9651	0.9014	0.9311	0.9153	0.8302	0.8996	2500 (Class 5)	Inactive	Inactive	Inactive	Inactive
Dukunolide B	2.09	Soluble	Low	Yes	No	0	148.33	No	0.9876	0.7312	0.7957	0.8360	0.7483	0.9308	555 (Class 4)	Inactive	Active	Inactive	Inactive
Chlorogenoquinone	0.22	Very soluble	Low	Yes	No	2	158.43	No	0.6350	0.7382	0.9477	0.8825	0.8960	0.9002	50 (Class 2)	Inactive	Inactive	Inactive	Inactive
Thamnosin	4.63	Poorly soluble	High	Yes	Yes	0	78.88	No	0.9907	0.8063	0.5228	0.6371	0.8185	0.8590	500 (Class 4)	Inactive	Inactive	Inactive	Inactive
Luteolin	1.86	Moderately soluble	High	Yes	Yes	1	111.13	Yes	0.9650	0.8957	0.9106	0.9025	0.7898	0.9116	3919 (Class 5)	Inactive	Active	Active	Inactive
Catechin	1.33	Soluble	High	Yes	Yes	1	110.38	Yes	0.9654	0.8956	0.9046	0.9041	0.8227	0.8771	10,000 (Class 6)	Inactive	Inactive	Inactive	Inactive

**Table 6:** Molecular docking grid box coordinators used by Autodock Vina.

Centre	x	y	z
Human HMG-CoA reductase (PDB ID:1HWK)	18.313098	8.379805	15.174463
Size	x	y	z
	12	12	12
Exhaustiveness	8		

## Data Availability

We analyzed existing, publicly available data. The accession numbers for the datasets are listed in the key resources table. Any additional information required to reanalyze the data reported in this paper is available from the lead author upon request.

## Abbreviations

AC	Atherogenic Coefficient
ADMET	Absorption, Distribution, Metabolism, Excretion, and Toxicity
ATV	Atorvastatin
AIP	Atherogenic Index of Plasma
AMR	Antimicrobial Resistance
ATP	Adenosine Triphosphate
CaCO_2_	Calcium Carbonate
CAT	Catalase
CK-MB	Creatine Kinase-MB
CRR	Cardiac Risk Ratio
CYP1A2	Cytochrome P450 1A2
CYP2C19	Cytochrome P450 2C19
CYP2C9	Cytochrome P450 2C9
CYP2D6	Cytochrome P450 2D6
ECG	Electrocardiogram
GSH	Reduced Glutathione
H_2_O_2_	Hydrogen Peroxide
HBA	Hydrogen Bond Acceptor
HBD	Hydrogen Bond Donor
HDL	High-Density Lipoprotein
HIA	Human Intestinal Absorption
HMG-CoA	3-Hydroxy-3-Methylglutaryl Coenzyme A
HR-LC–MS	High-Resolution Liquid Chromatography–Mass Spectrometry
iNOS	Inducible Nitric Oxide Synthase
ISO	Isoproterenol
LD_50_	LD_50_ is the abbreviation used for the dose which kills 50% of the test population
LDL	Low-Density Lipoprotein
logP	Logarithm of the Octanol-Water Partition Coefficient
MDA	Malondialdehyde
MI	Myocardial Infarction
MW	Molecular Weight
NCEE	*Neolamarckia cadamba* Leaf Ethanol Extract
PDB	Protein Data Bank
ROS	Reactive Oxygen Species
SGOT	Serum Glutamic Oxaloacetic Transaminase
SOD	Superoxide Dismutase
TC	Total Cholesterol
TG	Triglycerides
TPSA	Topological Polar Surface Area
VLDL	Very Low-Density Lipoprotein
